# Trends in the texts of national anthems: A comparative study

**DOI:** 10.1016/j.heliyon.2023.e19105

**Published:** 2023-08-11

**Authors:** Radu Silaghi-Dumitrescu

**Affiliations:** Institute for Interdisciplinary Studies on Bio-Nano-Sciences, Babes-Bolyai University, Cluj-Napoca, Romania

**Keywords:** National anthem, Feeling, Liberty, Statistical analysis, Language

## Abstract

In a recent previous investigation of national anthems, preferred topics and their bias (e.g., towards identity, fighting, or well-being) were identified subjectively (Silaghi-Dumitrescu, 2020). The present report aims to verify whether a more objective, automated, comparison of the texts of national anthems across the world can also reveal systematic trends – and to what extent. To this end, the Tropes and Semantria software packages are employed, revealing preferred topics (e.g., state, feeling, body, time, land, religion, family, fight), how their relative weights differ across continents and cultures, and how the conveyed sentiments vary. For instance, “liberty” is more common in Latin-country anthems while almost absent in Asia, “feelings” are less mentioned in Germanic-language anthems, and the first-person singular “I” is essentially absent African anthems. The sentiment scores of the anthems vary from neutral in Latin and Mediterranean anthems to much more positive in Central and Western Asian, Germanic and Slavic countries.

## 1 Introduction

Music lends itself to cross-cultural comparisons, having been shown to either signal social/group/race/national identity, or reinforce it, or contribute to its modulation/refinement, or act to modulate intergroup communication (either helping in conflict resolution or in its exacerbation) – ultimately with the potential of a social engineering tool both towards establishing identities and towards negotiating between groups of different/competing identities. Applying the framework of optimal distinctiveness theory (ODT), Abrams points out, based on studies on two groups of ∼50 and ∼2600 young individuals/students, that musical preferences are geared towards neither very individual nor very general choices, but rather intermediate ones, as expected based on ODT – where a balance between identity definition (at the limit, an individualizing trait) and inclusion (at the limit, a collective-related trait) is sought [1,2]. Musical preference-based favoritism within social groups was demonstrated, favoring stereotypies identified by group members as group identity-related [3]. In a study with ∼140 African American college students, Dixon et al. found that preference for rap music was correlated with increased self-esteem. Also, Afrocentrist attitudes were correlated with preferences for music featuring strong Afrocentric characters. Within the same group, preferences for music with misogynistic components was found to correlate with the belief that the respective music was not degrading towards women [4]. Related to this, Reyna et al. found that, in other social groups, negative attitudes toward rap music correlate with negative stereotypes of African Americans (e.g., laziness) [5]. In a study on 80 British young individuals, Rentfrow et al. showed that music preferences do form the basis of judgements/perceptions about psychological and social characteristics of individuals and groups [6]. In another example of identity-related type of music, i.e. the Gypsy-associated Flamenco music in Spain, respondents’ attitudes towards the Gypsy community were more positive if the Flamenco was invoked as a positive defining feature of that community; however, a similar music-identity manipulation could not be performed in relation to another minority – the North Africans [7]. Bensimon, in a study based on interviews with a small number of protesters and with their opposing security forces, reported that protesters were able to elicit empathy from security forces when resorting to slow/quiet/sad songs, but that the opposite effect was obtained with songs flaunting identity (be it national or religious, even though this identity would have been common between the protesters and the security forces) [8].

It has been argued that the lyrics of national anthems would also (if not more so) be suitable for exploring identity/group issues [9]. Cerulo has also showed that the musical tune structure of the national anthems is directly affected by social power relationships within the society at the time when the anthem is adopted [10]. Examinations of the lyrics of national anthems have to date included interpretations/commentaries on their nationalism, family, sexism, suicide rates and others – with cross-cultural implications in several cases. Gloominess, nationalism, gender-bias or other features were described in such analyses as features of some of the national anthems. The perceived relevance of such data has led to proposals going as far as replacing national anthems with more positively-oriented versions so as to reduce the rate of suicide in the respective nation [11]. In comparing 6 national anthems, Vörös et al. find that the anthems of countries with lower suicide rates tend to contain relatively more positive contents, emotions and intentions, while in the anthems of countries with higher suicide rates more ambivalence, denial, loss or even aggressive and self-destructive implications were found [12]. A critical stance towards national anthems per se is also documented (e.g., “glorified triviality”, “ridiculous pathos”) [13]. The need for contextualization and avoidance of absolute engagements especially in educational settings was advocated in case studies of the United States of America (USA) and Great Britain anthems. One may cite in this context for the USA controversies regarding the mention of slavery, or the fact that while the verses were composed to commemorate a victory against British military forces, the musical tune itself was British [[14], [15], [16]]. Winstone et al. pointed out a notable age dependence of the identity-building/modelling effect of the national anthem in a sample of children aged 8–10 in Great Britain [17]. A need for balance between the unifying role of a national anthem and the inherent conflicts engendered by dogmatic treatment/implementation thereof was also pointed out [15,18]. In a study on ∼100 secondary students from choirs in Canada and USA, Gilboa and Bodner found that Canadian students were significantly more proficient in performing the national anthem – suggesting that the social impact of the anthem is also significantly dependent on a range of factors, of which education is a key part [19]. In a study of citizens from various backgrounds within the same country, it was found that the national anthem evoked more national associations than any other songs considered. Interestingly, the degree of this trend did differ when marginal groups of the society were considered [20]. Kyridis et al. provided a comparison of 18 national hymns (Russia, Argentina, Austria, Mexico, Canada, Greece, Cuba, Bulgaria, Germany, F.Y.R.O.M., U.K., Serbia, Romania, Albania, Australia, U.S.A., France and Belgium) with emphasis on nationalistic, propagandistic and occasional chauvinistic features and on the degree of expressing identity/belonging [21]. Lauenstein et al. pointed out a focus of anthems on family as a background for promoting, hierarchical structure, social roles and responsibilities (with gender implications), and positive affective connotations [22]. Liao et al. point out, using the Chinese national anthem as a case study, an active role for national anthems in creating a collective memory – as well as the complex interplay with cognitive and social contexts [23]. A similar analysis was provided by Siska for the Turkish national anthem [24], or by Pavković for the countries originating the former Yugoslavia [25]. Oluga et al. pointed out inherent linguistic sexism in a series of ∼60 West/North Germanic and Romance/Italic language national anthems – but also pointed out how these traits were occasionally altered (either exaggerated, or attenuated) during translation into other languages [26]. In the same vein, Rodriguez identified notable distortions within the official translation of the Venezuelan national anthem from Spanish to the native language of Warao, with social, cultural and political implications [27].

The above-discussed studies on national anthem lyrics have focused on narrow sets of anthems or topics. It is thus unclear to which extent their conclusions are generally applicable to all anthems, and/or whether the previously-examined topics are the only ones, or even the dominant ones, in national anthems. Therefore, a more systematic analysis, bound to intrinsically uncover trends across nations/cultures [28,29], may be deemed useful. On the other hand, attempts towards cross-cultural analysis on a larger scale would have to be conservative and to consider the very different backgrounds of anthems in terms of their origins. One may consider for instance that some lyrics were written decades or centuries before becoming anthems, while some were written on purpose to this extent. Also, that some were validated by public popularity before the adoption of anthems, some afterwards, and some less so – especially in less democratic countries. Indeed, writing words for anthems is by no means a general institutionalized practice across the world, and naturally so in view of how rare the event of anthem writing is. If any general trends and correlations are to be identified, one would subsequently need to seek links with the poetic/literary field they emerged from and to the historical context thereof. Fluctuations of literary/poetic language in diverse traditions would then be of importance. Anthems could also be seen as an instance of selection in cultural production by political elites at the specific point in time (as already pointed out for the musical tunes thereof [10]). Thus, anthems may well not be expected to describe cross-cultural variation and any features of “nations” (e.g., elites could be global and copy each other or try to innovate to be different from neighbors), or correlate with current sociological data. An attempt to nevertheless perform a comparison of most of the known official national anthems across the world (∼200) was recently reported, focusing on identification of the pervasive topics, and on possible connections between these topics and some basic societal features [30]. A set of recurring themes was identified subjectively by the authors of the study, as follows: ancestry/past, beauty, build/work, country name, courage, democracy, enemy, ethnicity, family, man, woman, fight, flag/colours, forever/never, future, geographical references, glory, independence/freedom, joy/happiness, home/mother/father-land, law/governance, leader, love, loyalty, peace, poverty/wealth, pride, religion, revolution, sacred, sacrifice, salvation, sorrow, treason, tyrant/chains, unity, win/victory. The number of topics present in a given anthem, as well as their type were found to vary significantly between anthems. Groups of anthems were defined based on these tendencies. Correlations, albeit weak, with more general societal features such as age of country, gross domestic product (GDP) per capita, Gini coefficient, size of armed forces, inequality, inequality-adjusted human development index, and a number of parameters from the World Values Survey (WVS) database (related to religion, gender equality, attitude towards other nationalities/races, attitude towards work, attachment to democratic values etc.) were identified. A shortcoming of the study was not only its subjective character, but also its limited quantitative methodology: it did not differentiate between the number of times a given topic was mentioned in a given anthem. Among other things, a detailed evaluation of the sentiments conveyed by the anthem would be very difficult under these conditions (e.g., it would not differentiate between an anthem that mentions “sorrow” once in a total of 10 phrases, and an anthem that mentions the same notion in each of the 10 phrases).

The aim of the present study is thus to employ an objective methodology in comparing national anthems in terms of their content and message. An automated analysis is therefore reported, where the notions present in national anthems are indexed automatically using text analysis software, and their frequency of occurrence within each anthem is accounted for. This is the first time such a comprehensive *objective* analysis is performed on this body of texts. The objective character of the analysis is particularly important since national anthems are indeed crafted to elicit significant emotional/subjective/ideological bias. Previous studies, as discussed above, have focused either on narrow sets of anthems, or on researcher-chosen topics. With an automated/objective methodology, one may be expected to be able to more reliably assess (1) the dominant topics and the degree to which anthems adhere to previously-identified clichés about extolling national identity, (2) the degree of differences in cultures along these coordinates, (3) the correlations, if any, between numerical data derived from anthems and from other societal indicators. An added advantage of the automated analysis is that it also allows for statistical identification of parts of speech/syntax, thus extending the information from merely listing topics to also putting these topics into contexts. For instance, one may distinguish between texts that are predominantly descriptive and texts that are predominantly action-related, or between texts that put emphasis on collectivity and those that put emphasis on individuals. This line of study provides the premises (and background comparisons) for more informed subsequent analyses of each anthem or of groups of anthems. Thus, should one wish to analyze an individual anthem from the point of view of the sentiment score (negative vs. positive or neutral), it would be essential to know what the general/average values are in anthems in general across the world, as well as in anthems of countries sharing similar backgrounds (whether in terms of geography or in terms of type of language); likewise for the preferences in topics addressed by the respective anthem, or for syntax/grammar preferences. One could then assert whether any choices made by the anthem were intrinsic to the respective country independently due to exclusively internal factors, or whether they are a common feature dictated by cultural/regional factors also present in (and also affecting the anthems of) other countries.

## 2 Methods

### 2.1 Data collection

The English versions of the texts (with caveats pointed out e.g. in ([26,31]) were employed. Data concerning the anthems, including the English version of the text, were from Wikipedia.org (https://en.wikipedia.org/wiki/List_of_sovereign_states_by_date_of_formation, https://en.wikipedia.org/wiki/List_of_countries_and_territories_by_population_density, https://en.wikipedia.org/wiki/List_of_national_anthems, https://en.wikipedia.org/wiki/Category:Lists_of_wars_by_country, https://en.wikipedia.org/wiki/List_of_countries_by_inequality-adjusted_HDI), http://www.worldvaluessurvey.org and http://www.national-anthems.org/. The use of English translations puts a limit on the extent of data which can be meaningfully compared between texts originating in different languages. Detailed syntactic comparisons are hence not possible with this approach. Moreover, the small selected set of parameters which are analyzed in the present study will bear two different imprints: the intrinsic imprint of the historical and sociological context, and a linguistic one which may sometimes be difficult to separate from the latter. The types of analyses considered in the present text are therefore conservative and limit themselves to items that are expected to be conservable in translation – e.g., the types of nouns used, or the temporal setting, or the prevalence of verbs, or the types of verbs but not their exact identity, or the general feeling (positive vs. negative).

Anthems for 186 countries were considered initially, not including states that are currently in the process of international recognition and/or of establishing their independent structures. Detailed analyses regarding correlations between the anthem data and general objective numerical parameters of the country (GINI, population, GDP and others) were however performed only on a set of 145 countries for which such data were available. For a smaller number of countries, for which such data were available (51), correlations were also examined with parameters extracted from the World Values Survey database, wave 6: 2010–2014 (http://www.worldvaluessurvey.org). For these countries, correlations with the inequality-adapted human development index (IHDI) were also analyzed.

### 2.2 Data analysis

Anthems were grouped together as described in text (based on geographical regions, or to some extent on the type of original language, as described below) and analyzed using the Tropes software package, [32]. For each group of anthems, data was retrieved in automated manner (without intervention/bias from the user) about (1) the most abundant notions/references and (2) most abundant word categories – all three as defined within the software package without altering the standard settings. An exception from the standard settings was made for the most abundant notions/references, where the software generally identifies country names as belonging to categories named after geographical areas (e.g., Africa, Middle East, Oceania, Asia etc.); these categories are reunited under the common heading “country name” in Table 2. The Tropes software was developed by Pierre Molette and Agnès Landré on the basis of the work of Rodolphe Ghiglione (http://www.semantic-knowledge.com).

**Table 1:** Word categories in national anthems (proportions, %). Outliers are shown in bold.

Word category	W	Africa	Amer-Lat	Asia	Asia-CtW	Asia-SE	Eur	Eur-Balk	Eur-Ger	Eur-Latin	Eur-Slav	Eur-W	W-Medit	W-Eng
Verbs														
Factive	40	42	40	**36**	**32**	40	42	**44**	**45**	41	**34**	43	41	37
Stative	34	**27**	32	**45**	**55**	37	37	**42**	31	33	**48**	31	36	32
Reflexive	26	**31**	28	**20**	**14**	22	21	**14**	24	24	**19**	26	23	**31**
Performative	0	0	0	0	0	0	0	0	0	1	0	0	0	0
Connectors														
Condition	2	2	**9**	1	0	1	1	0	0	0	0	0	1	1
Cause	2	1	2	4	0	5	2	3	1	4	5	1	5	3
Goal	0	1	0	1	0	1	0	0	0	0	0	0	0	0
Addition	76	81	**63**	79	84	79	77	**45**	**88**	78	68	**85**	68	82
Disjunction	4	3	**10**	1	3	0	3	**8**	1	**11**	0	2	2	4
Opposition	4	4	6	2	1	4	3	**11**	1	4	5	2	4	4
Comparison	8	7	9	8	6	9	7	**16**	6	**4**	5	5	**14**	**2**
Time	4	1	3	4	7	1	7	**18**	2	0	**18**	5	7	5
Place	0	0	0	0	0	0	0	0	0	0	0	0	0	0
Modalities														
Time	34	33	33	38	**47**	**23**	33	29	**22**	**50**	28	32	33	33
Place	20	22	19	**14**	7	23	19	**14**	19	**13**	22	16	**14**	20
Manner	18	18	19	18	14	**25**	21	21	**31**	**8**	**26**	21	17	**10**
Assertion	2	2	0	1	0	2	2	0	3	4	0	5	3	4
Doubt	0	0	0	0	1	0	0	0	0	0	0	0	1	0
Negation	11	**5**	**17**	**16**	**17**	**17**	9	**23**	**2**	8	11	7	**21**	11
Intensity	16	20	12	13	14	**10**	16	14	**24**	17	13	19	12	22
Adjectives:														
Objective	59	59	**53**	60	62	62	62	61	62	62	63	62	**67**	57
Subjective	36	34	**43**	35	36	32	32	36	**27**	31	35	**29**	30	**41**
Numeral	5	7	4	5	2	6	6	3	**12**	7	2	**9**	3	2
Pronouns														
I	5	**0**	**0**	**12**	7	**18**	8	7	5	**19**	2	7	3	1
Thou	9	10	8	7	4	**17**	8	5	**12**	**0**	6	**12**	3	**18**
He/she	4	0	8	3	4	4	4	2	2	4	0	6	2	4
We	53	**69**	47	43	**40**	**40**	42	**36**	43	**39**	56	45	50	**66**
You	18	12	14	24	**34**	**8**	26	**34**	**30**	**31**	16	24	**32**	**5**
They	5	3	7	5	7	7	4	5	3	8	6	4	5	4
Somebody	0	1	1	0	0	0	0	0	0	0	0	0	0	0

**Table 2:** Relative usage of most common terms encountered in national anthems across the world. Normalized frequency is listed (fractions reflecting the number of occurrences of the respective term, compared to the number of occurrences of the most frequent term; for the world average, values down to 0.3 are shown).

terms	World	Africa	Amer-Lat	Asia	Asia-CtW	Asia-SE	Eur	Eur-Balk	Eur-Ger	Eur-Lat	Eur-Slav	Eur-West	W-Medit	W-Eng
country name	1.0	1.0	0.6	0.9	0.7	1.0	1.0	1.0	1	0.7	1.0	1.0	1.0	0.8
state	0.6	0.4	1.0	1.0	1.0	0.7	0.8	0.7	0.9	0.5	0.5	0.8	0.7	0.5
feeling	0.5	0.5	0.6	0.5	0.4	0.5	0.4	0.4	0.2	0.3	0.4	0.4	0.4	1.0
body	0.4	0.4	0.6	0.4	0.2	0.5	0.7	0.7	1.0	1.0	0.1	0.9	0.6	0.4
time	0.4	0.4	0.4	0.3	0.3	0.2	0.7	0.4	0.9	0.4	0.4	0.8	0.3	0.5
land	0.4	0.3	0.4	0.4	0.3	0.3	0.6	0.4	0.6	0.2	0.5	0.7	0.3	0.8
religion	0.3	0.3	0.2	0.4	0.3	0.2	0.5	0.4	0.5	0.5	0.2	0.8	0.3	1.0
family	0.3	0.3	0.5	0.2	0.2	0.2	0.6	0.4	0.4	0.5	0.5	0.6	0.3	0.5
fight	0.3	0.2	0.7	0.2	0.1	0.2	0.5	0.7	0.3	0.7	0.2	0.4	0.4	0.3
behavior	0.3	0.3	0.8	0.3	0.2	0.2	0.2	0.2	0.2	0.5	0.2	0.2	0.3	0.3
liberty	0.3	0.4	0.8	0.1	0.1	0.1	0.5	0.4	0.9	0.3	0.2	0.7	0.2	0.3
social group	0.3	0.3	0.4	0.4	0.3	0.4	0.2	0.3	0.0	0.3	0.2	0.2	0.2	0.2

Anthems were grouped based on geographical and linguistic categories as follows: World (W, all anthems), World-English (W-Eng, countries where English is an official as well as the major native language), World-Mediterranean (W-Medit, countries around the Mediterranean sea, from Europe, Middle-East and Northern Africa), Asia, Central and Western Asia (Asia-CtW, with limits set at China and India, not included), South-Eastern Asia (Asia-SE, countries not included in Central and Western), Africa (excluding northern/Mediterranean countries), Latin American (Amer-Lat; this is a geographical classification but may also be regarded as a linguistic one, since the vast majority of Spanish-language anthems reside in this region), Europe (Eur), Europe- Western (Eur-W, with eastern limits set to Italy, Switzerland, Austria, Germany, Sweden, Finland), Europe-Eastern (Eur-E, former members of the communist bloc), Europe-Balkan (Eur-Balk, Balkan countries including Turkey; this grouping overlaps to a good extent with the Slavic group, but on the other hand is justified due to historical reasons pertaining to a common heritage influenced by the centuries-long domination of the ottoman Empire in the region), Europe-Germanic (Eur-Ger, languages in the Germanic group excluding English language), Europe-Slavic (Eur-Slav). Where the frequency of words or notions across groups of anthems is examined, data are given in terms of relative frequencies within the group rather than absolute number of occurrences, so as to normalize against the distinctly different lengths of anthems and distinctly different number of countries in each group.

Sentiment scores were attributed for each anthem individually (not per group) using the web interface of the Semantria software package [33] without any further intervention or processing from the user, except for adding a space character at the end of each verse/line when pasting the text into the analysis window, in order to ensure word separation across lines. This automated analysis employs natural language processing (NLP) and machine learning techniques in order to identify sentiment-bearing phrases and/or components, and assign each of them a sentiment score ranging from ∼ −1 to 1 for simple expressions. A total score is assigned for the analyzed document by averaging the values for each of the sentiment-related word/expression. For instance in the case of the Romanian anthem, the automatically-recognized sentiment-related terms were “slaves” (score −0.929), “deadly”, “barbaric”, “death”, “tyrants” (scores of −0.600 each), “weapons”, “enemies” (−0.490 each), “renew” (+0.450), “great” (+0.600), “freedom” (+0.680), “holy” (+1.000), “glory” (+1.145), “victor” (+1.503), “pride” (+1.800) – averaging to a total score of 0.205 for the Romanian anthem. Within the framework of the Semantria feeling analysis, texts with total scores above 0.250 are considered to have a positive overall sentiment; texts with total scores between 0.250 and −0.150 are assigned as neutral, while those below −0.150 are categorized as displaying an overall negative sentiment [33].

Averages of sentiment scores for each group of anthems were calculated either based directly on these values, or in a weighted manner taking into account the relative populations of the respective countries.

All calculations were performed within a Microsoft Excel spreadsheet with standard formulae for averages and correlation coefficients.

## 3 Results and discussion

### 3.1 Relative frequency of word categories

Table 1 shows the most common categories of words encountered in national anthems. Verbs expressing actions (“factive” in Table 1) are less common in Central and Western Asian, (32%) vs. the world-average of 40%. Verbs expressing states and possession (“stative” in Table 1, 34% world average) occur less often in Africa (27%) but distinctly more in Central and Western Asia and in Slavic anthems (48–55%).

Connectors are generally present in constant proportions across anthems, with a few exceptions. Thus, Latin American anthems are higher in conditional connectors (9%, vs. 0–1% in all the other groups). Addition connectors are more common in Germanic and Western European anthems (88% vs. a 76% average), but less common in Latin American anthems (63%). Disjunctions are more common in Latin and Latin-American anthems (10–11% vs. a world-average of 4%). Comparisons are more common in Balkan and less so in English-language anthems (16% vs. 2%, compared to a world-average of 8%). Time connectors are distinctly more common in Slavic anthems (18% vs. the world-average 4%).

Among the modalities/adverbs, those referring to time are distinctly more common in Central/Western Asian and Latin European anthems (47–50% vs. a world-average of 34%) and less so in South-East Asian and Germanic ones (22–23%). For place modalities, Asian, Balkan, Mediterranean, and Latin European show the smallest contributions compared to the average (13–14% vs. 20%). For manner modalities, English and Latin European anthems show smaller contributions (8–10%), with higher percentages seen in Germanic anthems (31%), compared to a world-average of 18%. Negations are less common in Germanic anthems (2%) and distinctly more common in Balkan and Mediterranean ones (21–23%), compared to the world-average of 11%. Intensity-related modalities are most common in Germanic and least common in South-East Asian anthems (24% and 10%, respectively, vs. an average of 16%).

Among adjectives, outliers are seen for objective (more common in Mediterranean anthems – at 67%, compared to an average of 59%), subjective (less common in Germanic– 27% vs. an average of 36%) and numeral ones (more common in Germanic ones −12% vs. an average of 5%).

The first-person singular “I” is essentially absent in African and Latin American countries but distinctly above average in South-East Asia and Latin Europe (12–19% vs. an average of 5%), while somewhat the reverse is true about the plural “we” (66–69% in African and English-speaking anthems, 36–43% in Asian, Balkan and Latin European anthems, vs. a world average of 53%). The second-person “thou” is expectedly more present in English-speaking anthems. When counting “thou” and “you” together, South-East Asian (12%) show a distinctly lower frequency than the world-average (27%), while on the opposite end are Central and Western Asia, Balkan, and Germanic anthems (38–42%). The third-person masculine pronoun “he/she” has a relatively low occurrence, and variations across groups is likely to not be statistically significant (though one may note the zero values in African and Slavic anthems). The term “he” appears 15 times throughout the anthems, whereas the feminine “she “appears only 6 times; whereas the masculine generally refers to the citizen, the feminine generally refers to the country – so the gender imbalance is even stronger than suggested by the 15:6 ratio. This expected patriarchal cliché is partly confirmed by the fact that the forms “him/his” appear for a total of 48 times, while “her” appears for 58 times – but almost all of the 58 times involve impersonal notions (liberty, victory, or, most often, the country) rather than persons (with the notable/famed exception, obsolete at the time of revising this text, of the British “God save the Queen”).

To sum up, Balkan anthems stand out with higher percentages of comparisons, negations, and second-person “you/thou”, as well as lower incidence of first person plural “we”. All of these may be argued to reflect an aspiration/inclination towards actively seeking differentiation against “others” – in line with the Balkan's XIXth century reputation as “the powder keg of Europe” and with the still extant ethnic and religious diversity [34,35]. These considerations may be illustrated by two examples – the Albanian and the Romanian anthems.

The Albanian anthem offers examples of negations (“not” and “don't”) and of comparisons (“as”), and no mention of “you/thou”; although on average the Balkan anthems make more use of “you” and less use of “we”, the Albanian anthem is an exception:

Around the flag united // With a desire and a reason // All vowing to him // To unite the word for the freedom // From the war the only one that gets away // Is that one that is born as a traitor // Who is a man is not scared // Dies, dies as a martyr // We will keep the arms in our hands // To protect our homeland in anywhere // Our rights we don’t share them // Here the enemies don’t have a place // The Lord Himself has said // That Nations vanish from the earth // But Albania will live // For her, for her we fight for.

The Romanian anthem also shows negation (“never”) and comparison (“better/than” – at 17% vs. an 8% world-average) and uses the second-person pronoun “you” (in fact, the entire line of speech is addressed in second-person to the “Romanian”); the plural “we” is present, though it remains less common than in other anthems (as one can see by comparison with African anthems below):

Wake up, Romanian, from your deadly sleep//Into which you've been sunk by the barbaric tyrants//Now, or never, your fate renew,//To which your enemies will bow.//Now or never let's give proof to the world//That in these veins still flows a Roman blood,//That in our chests we still maintain our pride in a name//The victor in his battles, the name of Trajan!//Watch on, shadows of highnesses, Mihai, Stefan, Corvinus,//The Romanian Nation, your great grandchildren,//With weapons in their arms, with your fire in their veins,//“Life in freedom or death!” shout all.//Priests, lead with your crucifixes! Because our army is Christian,//The motto is Liberty and its goal is holy,//Better to die in battle, in full glory,//Than to once again be slaves upon our ancient ground!

Germanic anthems stand out with higher percentages of addition connectors, manner and intensity modalities, numerals and the use of “you/thou”, and also with lower incidence of negations, subjective adjectives, and time modalities. The German anthem illustrates these observations to some extent, with repeated use of the addition connector “and” (5 times, compared to 1 in the (longer) Albanian and Romanian anthems), the use of modalities (“brotherly”), no negations whatsoever, no mention of time, no subjective adjective:

Unity and justice and freedom//For the German fatherland!//For these let us all strive//Brotherly with heart and hand!//Unity and justice and freedom//Are the pledge of fortune;//Flourish in this fortune's blessing,//Flourish, German fatherland!

The Icelandic anthem, with its repeated use of “thousands”, is largely responsible for the high average of numerals among the Germanic anthems. It also makes repeated use of the addition connector “and” (9 times), manner modalities (“reverently”, “safely”), and again zero negations.

Central/Western Asian anthems stand out with more time-related adverbs and less action-related verbs. The Afghan anthem may exemplify this, by having no factive verbs while indeed making reference to the time modalities (“for ever”). The Tadjik anthem also illustrates these two trends – with a low percentage of action-related verbs (“give”, “reach”, “stand: less than a quarter of all verbs) and with repeated use to time modalities (“long live”, “forever”):

Our beloved country, // We are happy to see your pride. // Let your happiness and prosperity be forever. // We have reached this day since ancient times, // And we are standing proudly under your flag, under your flag. // Long live my homeland, // my free Tajikistan! // You are a symbol of our ancestors' hope // Our honour and dignity,

You are an eternal world for your sons, // Your spring will never end, // We shall remain loyal to you, loyal to you. // Long live my homeland, // my free Tajikistan! // You are a mother for all of us, // Your future is our future, // Your meaning is the meaning of our souls and bodies, // You give us happiness forever, // Because of you, we love the world, love the world! // Long live my homeland, // my free Tajikistan!

South-Eastern Asian anthems feature less intensity or time modalities, and of “you/thou”. The Bangladesh anthem, despite its relative length, indeed has zero intensity adverbs and only two instances of time-related ones (“forever” and “at once”); it does on the other hand use the pronoun “you” more than other SE Asian anthems - though distinctly less than the pronoun “I”. A second SE Asian anthem may be invoked along these lines – that of Vietnam. Indeed, here there are no time or intensity modalities, and no use of “you/thou”:

Armies of Vietnam, forward!//With one single determination to save our Motherland,//Our hurried steps resound on the long and arduous road.//Our flag, red with the blood of victory, bears the spirit of the country.//The distant rumbling of the guns mingles with our marching song.//The path to glory is built by the bodies of our foes.//Overcoming all hardships, together we build our resistance bases.//Ceaselessly for the People's cause let us struggle,//Let us hasten to the battlefield!//Onward! All together advancing!//For one eternal Vietnam.

Slavic anthems stand out with higher incidence of stative verbs (expressing states or possessions) and time connectors. The Polish anthem has a 38% predominance of stative verbs (“be”, “seem”, “live”) vs. the 34% world-average, and is also a rare example of an anthem that employs a time connector (“so long as”):

Poland has not perished yet // So long as we still live // What foreign force has taken from us // We shall take back with the sword. // March, march, Dabrowski // From Italy to Poland // Under thy command // Let us now rejoin the nation // Cross the Vistula and Warta // And Poles we shall be // We’ve been shown by Bonaparte // Ways to victory // March, march … // Like Czarniecki to Poznan // After Swedish occupation, // To rescue our homeland // We shall return by sea // March, march … // Father, in tears // Says to his Basia // Just listen, it seems that our people // Are beating the drums // March, march …

The Russian anthem, as a second example of Slavic anthem, offers an even stronger example of high prevalence of stative verbs (75% vs. other types of verbs, more than double compared to the world-average) – though it does not use a time connector.

African anthems display higher percentages of plural first person pronouns - and lower percentages of first person singular pronouns. Of all the groups of anthems, the African ones may be interpreted to most clearly reflect an aspiration towards group identity and unity, perhaps in a collectivistic sense [36]. The anthems of Zimbabwe and Mali (the former listed below), illustrate this state of things – zero use of “I” but repeated use of “we”:

Oh lift high the banner, the flag of Zimbabwe // The symbol of freedom proclaiming victory; // We praise our heroes' sacrifice, // And vow to keep our land from foes; // And may the Almighty protect and bless our land. // Oh lovely Zimbabwe, so wondrously adorned // With mountains, and rivers cascading, flowing free; // May rain abound, and fertile fields; // May we be fed, our labour blessed; // And may the Almighty protect and bless our land. // Oh God, we beseech Thee to bless our native land; // The land of our fathers bestowed upon us all; // From Zambezi to Limpopo // May leaders be exemplary; // And may the Almighty protect and bless our land.

Latin American anthems have higher incidences of conditional connectors - and lower incidence of singular first person nouns and additions. An example may be the anthem of Mexico (listed below) – which does use a conditional connector (“if”, while the world-average use of these is very close to zero), while not using “I” at all; it also makes less use of additional connectors (“and”; at 60% among connectors in this anthem, vs. 76% world average). A second example of Latin American Anthem may also be given – that of Brazil, again with zero use of “I”, a repeated use of “if”, and a lower-than average use of “and” (∼60%).

Mexicans, at the cry of war, // make ready the steel and the bridle, // and the earth trembles at its centers // at the resounding roar of the cannon. // and the earth trembles at its centers // at the resounding roar of the cannon! // Let gird, oh Fatherland, your brow with olive // by the divine archangel of peace, // for in heaven your eternal destiny // was written by the finger of God. // But if some enemy outlander should dare // to profane your ground with his step, // think, oh beloved Fatherland, that heaven // has given you a soldier in every son.

Latin European anthems display more time modalities - and fewer manner and place modalities. An apparent relative high incidence of “I” seen in Table 1 is in fact an artifact due to the small size of the analyzed set of anthems (the Andorran anthem has an 80% prevalence of “I”, while the other ones have 0% prevalence). An example of Latin European anthem is the Romanian one, listed above. There, one may indeed see repeated use of time modalities/adverbs (“now”, “still”, “once again” – at 50% of all adverbs vs. a 34% world-average), and less manner and place modalities (only one mention – “in full glory”). A second example may be taken that of the Spanish anthem, which does highlight its time modalities (“long live”) – though in this case the place and manner ones are rather on the average than below the world-average:

Long live Spain! Let's sing together, with different voices, and only one heart. // Long live Spain! From the green valleys, to the immense sea, a hymn of brotherhood. // Love the Fatherland, which knows how to embrace, below its blue sky, people in freedom. // Glory to the sons who have given to history justice and greatness, democracy and peace

The anthems of English-language countries display higher incidences of first-person plural pronouns and fewer manner modalities and comparisons. The Irish anthem (listed below) is such an example (“we” is the only pronoun present, there are zero manner modalities, zero comparisons). The United States of America anthem likewise has a high incidence of “we” (two thirds of the pronouns in this text) and zero incidence of comparisons – though the manner modalities do remain close to the world average (∼20%).

Soldiers are we, // whose lives are pledged to Ireland, // Some have come // from a land beyond the wave, // Sworn to be free, // no more our ancient sireland, // Shall shelter the despot or the slave. // Tonight we man the “bearna baoil”, // In Erin’s cause, come woe or weal, // ‘Mid cannon’s roar and rifles' peal, // We’ll chant a soldier’s song.

Overall, the above considerations reveal clear differences in the manners in which various cultures address the anthems. These differences can be further explored if one examines the correlation coefficients between the percentages for each of the categories in Table 1, across the various groups of anthems, as shown in Supporting Information. Considering the small differences seen between most of the parameters in Table 1, these coefficients are all at ∼0.9 and above. The weakest correlations (0.81–0.89) involve Central and Western Asia vs. English-speaking countries, Slavic vs. Latin European, and the Balkans vs. several other groups (Africa, SE Asia, Germanic, Latin European, Western European, English). Most of these differences can be explained by the inherently different grammar/syntax structures of the respective languages.

### 3.2 Most common notions

The most common terms encountered in anthems across the world are shown on Table 2, alongside differences in frequency of usage of the respective terms in the anthems pertaining to the various groups. The nature of these terms on average across the world is expected considering the purpose of the anthems – i.e., the state/nation/country and its contents/definition (land, people, social structures/groups, religion, as well as liberty when asserting one's country in relation to the rest), allegiance/feelings towards it (this includes the engagement to “fight” as well as the term “body” in Table 2 – where the chest, arms, blood are often invoked).

The *country name* is expectedly the most common nation in most anthems worldwide. Latin America stands out with a distinctly lower ratio (0.6 compared to the world-average of 1.0) in this respect; ratios lower than 1.0 are also seen in Central and Western Asia (0.7, reflected in an overall 0.9 fraction for the total of Asia), Latin Europe (0.7), and for English-speaking countries (0.8). The reasons why an anthem would not include the name of the country may vary. The lyrics of some anthems precede the establishment of the country in its present form and were not written with the express intent of serving the purpose of national anthem. For instance, in the case of Romania (in Latin Europe) the lyrics date back to 1848, at a time when the Romanian territory was split into provinces or principalities that did not include the word Romania (nor variations thereof) in their names; it was only in 1859 that an entity named the United Romanian Principalities emerged, transformed decades later into the Romanian Kingdom, then various forms of names including the notion of Republic – until the current name, Romania, was adopted in 1990. In other cases, omitting the name of the country may be a matter of literary style and/or of the country name not being amenable to lyrics (e.g., for the United Kingdom of Great Britain and Northern Ireland).

Correlated with (and to some extent complementing) the country name, the *state* is the second-most common notion in the anthems – since it is precisely the state/country that the anthem is meant to describe and support. Exceptions may be noted for Africa and for English-speaking countries, where the frequency of this term is lower by +50% compared to the world average. This may be accounted for by the fact that most of the respective countries are newer and have had less time to identify themselves with the state than with other, more subjective and less institutionalized, defining features. To illustrate these considerations, one may examine the same examples of anthems as in the previous section. Thus, in the two African anthems (Mali and Zimbabwe), or in the two English-language ones (USA and Ireland) there are no references to the State (e.g., “state”, “country”, “motherland”, “nation”, “realm”).

*Feelings* are the second most common term in anthems across the world. The frequency of this term is smallest in Germanic anthems (by ∼70% compared to the world average) – perhaps in line with general public perceptions about the respective nations as well as in line with numerical data based on more general analyses of literature in a small set of languages [31]. To illustrate these trends one may look at the two Germanic anthems cited above (Germany and Iceland) – where feelings (e.g., love, admiration, pain, horror, as seen in Latin American anthems for instance) are not explicitly mentioned. The ratio between the use of state and of feeling is only slightly larger than 1 in the world average (0.6 vs. 0.5); intuitively, this may be expected since most anthems would logically speak of love/pride for the country. This trend is mirrored in several regional categories, but there are also severe exceptions. A slight reversal is seen in Africa (0.4:0.5), and a major one in English-speaking countries (0.5:1.0); both of these categories are dominated by relatively younger countries, where arguably the state is/was not yet an established a notion as it was in the older or more traditionally established countries. Indeed, a large *state:feeling* ratio is seen in Germanic anthems (0.9:0.2) and in Central and Western Asia (1.0:0.4).

Anthropomorphic *body*-related items (blood, chest, arms etc. – in principle partly related to individualization) are the third most common notion on average across the world. Slavic and Central/Western Asian anthems stand out with decreases of 70–90%.

*Time* is the fourth most-common topic; the Germanic anthems stand out with an increase of 60% over the world average. Indeed, in the Icelandic anthem time is the most common type of notion found (with repeated uses of “years”, “days”, “evening”, “eternity”) – though Germany does not explicitly mention time-related notions (of the 9 Germanic anthems, 5 nevertheless do).

For the fifth most common term, *land*, Central/Western Asia and Latin Europe show decreases of 50–65% over the world average. Indeed, when looking at the Central/Western Asian anthems, only 11 out of the 23 anthems do not mention this notion – with the above cited Tajikistan as an example; Afghanistan is among these countries the one with most mentions of “land”. Among the Latin European anthems, only three mention “land”; of the two examples whose text was given/analyzed above, Romania does so once (“ground”), while Spain does not.

*Religion*, the sixth most common topic, shows distinctly wider variations. Thus, it is much more common in English-speaking anthems (by 70%), and much less in Latin American, SE Asian, and Slavic anthems (by 55–65%). The two English anthems cited above do not reference religion; in fact, ∼half of the references to religion/divinity in this group of anthems comes from only two contributors with excessive use of the term (United Kingdom with “God save the Queen”, and New Zealand). Of the groups that show lower incidence of religion, the examples given above for Latin America (Mexico, Brazil), SE Asia (Bangladesh, Vietnam), and Slavic anthems (Poland, Russia) indeed make essentially no reference to religion.

*Family* (seventh most common topic) is distinctly less often invoked in Asian anthems compared to the rest of the world. Of the four Asian anthems cited above, three (exception – Vietnam) do mention family-related notions, but not as central ones. In fact, of the 43 Asian anthems examined here, only 11 make reference to family.

*Fight* (eighth most common topic) is distinctly under-represented in African, Asian and Slavic anthems (50–75%). Indeed the two African anthems cited above do not refer to fighting. Of the Asian ones cited as examples, Afghanistan makes one reference to a fight-related item (“sword”), Vietnam 4 (“guns”, “armies”, “battlefield”, “struggle”), while Bangladesh and Tajikistan have none. Of the 15 Slavic anthems analyzed here, 10 do not refer to fight-related terms (Russia included); Poland is an exception, but even there the anthem is centered more around preparation/marching than around fighting itself. At the other extreme, Latin America, Latin Europe and the Balkans stand out with the highest incidence of this notion (0.7, vs. 0.3 for the world average).

*Behavior* is over-represented in Latin America and to some extent in Latin Europe (0.8 and 0.5, respectively, vs. a world-average of 0.3). For Latin America, this does match the trend in *feelings*, where this group of anthems also had the highest incidence.

*Liberty* is underrepresented in Asian anthems (slightly more so in Central and Western Asia, −87%) and oppositely so in Latin American and Germanic (and implicitly to some extent Western European) ones (+80–90%). Indeed, out of the 43 Asian anthems, only 8 refer to liberty/freedom (and the four examples cited above, Afghanistan, Bangladesh, Tajikistan, Vietnam, are not among them). The two Latin American anthems cited above (Mexico and Brazil) make little reference to liberty (Brazil does so, Mexico not); in fact, only 9 out of the 20 anthems in this group do refer to liberty – though they do so extensively (in fact, Argentina, Brazil and Uruguay account for ∼ half the total mentions of liberty in the Latin American anthems; this is not surprising given the iterative appearance of the word in e.g. the Argentine anthem – “Freedom! Freedom! Freedom!“). Also, in the group of Germanic anthems, the German anthem does mention this notion but Iceland does not; in fact, responsible for the high apparent incidence of liberty in Germanic anthems is Belgium, with 18 out of the 23 mentions of liberty. To that extent, if one leaves out Belgium (arguably justified, as it is half-Latin), the remaining Germanic anthems have a particularly low incidence of the mentions of liberty.

*Social groups* (e.g., “people”, “tribe”, “group”, “orphans”, “leaders”, “elders”) are essentially absent from Germanic anthems (−100%) and also more generally in European anthems, as well as in the English-language ones. These terms are indeed absent from the German and Icelandic anthems, as well as from the English-speaking anthems of USA and Ireland.

Correlation coefficients between the groups of anthems for the data shown in Table 2 (cf. Supporting Information Table S2) vary widely, from 1 to −0.2 – significantly more so than for the types of words discussed in relation to Table 1, and expectedly so given the wider differences between percentages in Table 2 (again, expectedly, since these are more specific/detailed categories than those in Table 1).

Reasonable correlations with the world average are expected for all of the regions/categories of anthems; this is indeed the case especially for Asian and Mediterranean anthems (r = 0.9 cf. Table S2), and reasonably so for Europe and Africa (r = 0.7). At the opposite end, English-language anthems only show an r = 0.3 vs. the world average, followed closely by Latin Europe at 0.4. Moreover, the English-language anthems show negligible correlations towards any of the other groups of anthems in Table S2.

African anthems correlate very little with those from other regions, with the smallest correlation, r = 0.0 for Latin Europe followed by Western Europe at 0.1. These are regions of the world that for a long time exerted political and cultural dominance over Africa, and from which African countries have been indeed seeking to gain independence and delimitation; better correlations are seen for Africa against Latin America and Asia (∼0.5), suggesting that the magnitude of these correlations is not related only to the degree of cultural differences.

Latin American anthems also show their weakest correlations against Latin European and Western European anthems (0.0–0.2, i.e. essentially absent), despite the historical and cultural connections between the two groups of nations. Against the background of a 0.95 correlation coefficient based on the structural grammar data in Table 1, these numbers suggest that a common language or even common history can still leave significant room for cultural differences that may outgrow the differences towards cultures of other languages.

The two halves of Asia examined in the present study show reasonably high correlation coefficients against each other (∼0.8), despite their widely different language families and cultures. Their weakest correlations are with Latin Europe and English language (0.1–0.2, i.e. negligible), with distinctly higher values for Slavic and Mediterranean regions (0.5–0.6).

The Slavic anthems show distinctly lower correlations with the other groups of European anthems (down to −0.2 for Latin Europe), but somewhat better (though still small, 0.5) correlation with Central and Western Asia.

The Latin European anthems show very low correlation with the other groups – except for those where some geographical overlap exists (e.g., Mediterranean and Balkan, at r = 0.7).

The Balkan anthems show a reasonable correlation with Western-European data (∼0.8) and essentially none with the Slavic anthems (∼0.2). The latter is of note, since a good part of the Balkan countries speak Slavic languages. Again, historical and regional factors appear to offer distinct ground for cultural differences even between nations of identical or similar languages.

In a previous analysis [30], a list of topics was identified subjectively/manually in the same set of national anthems as analyzed here. The topics were ranked based on the number of anthems where they occurred, but the number of occurrences in each anthem was not counted. By contrast in the present study the topics were identified in automated manner and the number of occurrences within the text was also counted. With these methodological differences in mind, one may nevertheless compare the topics ranked in Table 2 with those from the previous study, noting the general similarities but also some differences. The previous study listed (in this order) the following as most common topics: land, future, country name, independence/freedom, pride, loyalty, geography, religion, unity, forever/never, ancestors/past, family, love. Indeed, “land” is also found among the most common topic in the present quantitative analysis cf. Table 2; “geography” does not appear as individual term in Table 2 but may be viewed as a more general proxy of “land”. “Future”, “forever/never” and “past” are not found by themselves in Table 2, but are arguably still encompassed by the 4th most common term there – “time”. “Country name” is identifiable in Table 2 as the top notion – “state”. “Independence/freedom” is identified in Table 2 as “liberty”. “Pride” and “love” are not present per se in Table 2, but may be taken as part of the second most common notion there, “feelings”. “Religion” and “family” are present in exactly the same form in Table 2. “Loyalty” and “unity” are notably absent from Table 2, unless they are deemed to be convoluted into other terms (e.g., “feelings”). There is thus an overall agreement between the results of the two methodologies. The case of the two terms, “loyalty” and “unity”, which appear to be among the top 10 notions in anthems according to the previous study [30] in terms of a binary count (are present vs. are not present), but are not identified among the top 10 notions in the present statistical study where the number of occurrences within each text is also counted (i.e., non-binary), may be instructive to note. On one hand, may interpret this difference as a manifestation of the subjective methodology in the previous study. However, one may also use this example to raise the question of the intrinsic relative weights of the words – and point out that by no means these are (or should be taken as) uniform, neither within the same culture and especially nor across cultures.

Table 3 summarizes the salient features identified for each class of anthems in comparison with the others.

**Table 3:** Main distinctive features of anthem groups identified from the current automated analyses. Separate rows for Europe and for Western Europe are not shown, as these categories did not stand out with anything compared to the other European ones.

Group	Distinctive feature		Least correlated with
	More of	Less of	
Africa	verbs expressing states and possession; *we*; *feeling:state* ratio	*I, he/she, fight*	Balkans, Central/Western Asia (syntax), Latin Europe (topics)
America-Lat	conditional connectors; *fight, feelings, behavior, liberty*;	additional connectors; *religion, country name,* sentiment score	
Asia		place modalities/adverbs; *we, family, fight, liberty*	Latin Europe and English-language anthems
Asia-CtW	verbs expressing states and possession, time modalities/adverbs; *you/thou*; *state:feeling* ratio; positive sentiment	verbs expressing actions; *country name*, *land*, *body*, *liberty*	
Asia-SE	*I*	time and intensity-related modalities/adverbs; *you/thou*; *religion*;	Balkans (syntax)
Europe-Balk	comparisons, negations; *you/thou*; *fight*	place modalities/adverbs; *we; positive* sentiment	all other groups (syntax, topics)
Europe-Ger	addition connectors, manner and intensity modalities/adverbs; numerals; *you/thou*; largest *state:feeling* ratio; *time*; positive sentiment	time-related modalities/adverbs, negations; subjective adjectives, *feelings, liberty, social groups;*	
Europe-Latin	time modalities/adverbs; *I, fight, behavior*	place and manner modalities/adverbs; *you/thou*; *country name, land*; positive sentiment	rest of the world (topics)
Europe-Slav	verbs expressing states and possession, time connectors; positive sentiment	*he/she, body*, *religion, fight*	Latin European (syntax)
World-Medit	place modalities, negations, objective adjectives	positive sentiment	
World-English	*we*; *religion;* highest *feeling:state* ratio	comparisons, manner modalities, *country name, state, social groups;* positive sentiment	Central and Western Asia (syntax)

While this body of data may offer grounds for further socio-cultural comparisons, with theoretical frameworks ranging from philosophy to exact sciences [[36], [37], [38]], further discussion about the cultural relevance of the anthem analyses must take into account the at-most-indirect representability of these texts for the respective nations. Though most of them were validated at least tacitly in time by the people of the respective countries, the anthems were generally adopted by a very small group of people (“elites”), often entirely unrepresentative, from a statistical point of view, of the social/demographic fabric of the country – and in many cases not even elected democratically to do so. One must thus consider in each case whether the texts chosen by these elites were describing the fabric of their nations, or the future goals thereof, or if in fact there was a hiatus between the respective elites and nations on these topics. The latter may be particularly relevant for anthems in countries that have more recently gained their independence (and hence where popular confirmation of the anthem over time is not a factor to be considered yet). Last but not least, in interpreting statistical data on the presence of types of words and notions in anthems belonging to various cultures, one must also point out that the intrinsic weights of various words/notions are not inherently uniform within the same culture and much less so across cultures. If one mentions “unity” and “time” with a similar frequency in a text, it should not follow that the importance received by the two terms in the mind of the citizen/speaker is equal – nor that their relative weights would be identical regardless of language, culture, or historical context. Because of this, the discussion of the present data is for the moment left at a rather basic level, since follow-up studies would need to entail a much more interdisciplinary approach – e.g., psychological, historical, social beyond the obvious linguistic and literary components.

### 3.3 Sentiment analysis

Sentiment scores were also attributed to each anthem, after which averages were calculated across regions; here, weighted averages were also calculated (perhaps relevant in regions with distinct differences between the sizes of the countries - e.g., India and China in SE Asia). Individual anthems were found to have scores ranging from ∼-1 to ∼+1, with a world-average of ∼0.4. Latin-European and English-language anthems are at the lower end of the positive scale – and in fact fall below the 0.250 threshold for the positive scores when computing the weighted average considering the sizes of the respective nations. Balkan and Latin American anthems are close to the threshold, too. At the other end, the most positive anthems are those of the Germanic, Central/Western Asian and Slavic nations.

**Fig. 1:**
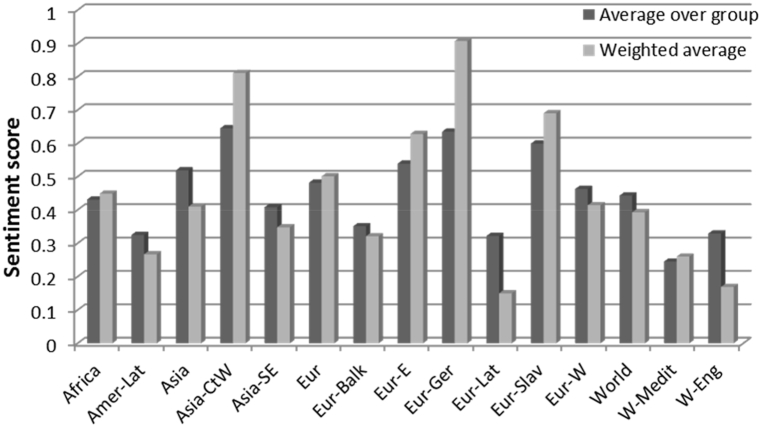
Sentiment scores for groups of anthems analyzed in the present study.

It may be noted that the averages listed in Fig. 1 have relatively large standard deviations (generally around 0.3, cf. Supporting Information Fig. S1 and Table 2). In Africa, the most notable outliers are Senegal and Central African Republic (which display negative values in a group where the average is distinctly positive), as well as Ghana and Niger (standing at twice the average positive score of the group). In Latin America, Peru is the sole notable exception, some 0.6 units more negative than any of the other anthems. In Asia, three countries -China, Japan and Vietnam - stand out with distinctly lower scores than the rest of their group. Central and Western Asia is on the other hand the most homogeneous group. By contrast, the Balkans offer one of the most heterogeneous set of values – spread across a full 1.5 units, twice as large compared to Central and Western Asia (with Bosnia & Herzegovina at the higher end at ∼ 1, and Albania at the lower one at ∼ -0.3). Among the Germanic anthems, Germany itself is and exception, with a score almost twice as positive compared to the average of its congeners from the same group (and in fact the third largest in the world, surpassed by only Georgia and Bosnia & Herzegovina). Among the Slavic anthems, the two southernmost Balkan nations of Montenegro and Northern Macedonia are by far outliers, with lower values compared to the rest. Western Europe is slightly more heterogeneous than the Balkans, with Ireland providing the most negative value against Germany's most positive. The Mediterranean region, more than others, appears split across two main relatively homogeneous subgroups: one with distinct positive values (e.g., Croatia, Egypt, Israel, Jordan, Morocco, Spain) and one with neutral and negative values (Albania, Algeria, France, Greece, Italy, Northern Macedonia, Montenegro).

The herein reported general positive-sentiment trend in national anthems is in line with the previous observation that human (written) language has a positive bias [31]. In the same previous study, it was also noted that among the ten languages studied, Spanish and Portuguese appear to have a higher positive bias than others – with Chinese, Korean and Russian at the opposite end. This is in contrast with the opposite finding in Fig. 1 for Latin-American (Spanish and Portuguese-speaking nations) and Russian, but in reasonable agreement for SE-Asian group. The case of English language is also illustrative: Dodds and co-workers find English books to be at the higher end of the positive bias scale defined by all languages studied, while English music lyrics are at the opposite end. Indeed, the anthem data of Fig. 1 correlate with this latter finding – and this may also offer at least a partial explanation for the Latin-American and Russian cases [31].

Possible correlations between the individual scores of each anthem, or the qualitative assignment (positive, neutral or negative), and a range of other parameters for the respective countries (size, age, size of armed forces, GDP, human development index, number of wars fought, World Values Survey (WVS) parameters) were then explored. No significant correlations were found for the countries where such data were available, except for a few weak cases. Correlations were found between the sentiment score and the number of wars fought normalized against the age of the country (0.4), and especially the number of wars lost (0.5). From the WVS parameters, the degree of trust in other nationalities (trusted completely or trusted somewhat) shows a 0.4 correlation with the qualitative sentiment assignment of the anthem.

Correlations between the individual scores of each anthem, or the qualitative assignment (positive, neutral or negative), and a range of other parameters previously collected for the respective countries (size, age, size of armed forces, GDP, human development index, number of wars fought, World Values Survey (WVS) parameters) were also explored. The respective correlation coefficients were generally very small. The largest values were for the correlations between the sentiment score and the number of wars fought normalized against the age of the country (0.4), and also against the number of wars lost (0.5). From the WVS parameters, the degree of trust in other nationalities (trusted completely or trusted somewhat) showed a 0.4 correlation with the qualitative sentiment assignment of the anthem.

When examining the correlations between the averages of the respective values per group of anthems, rather than between all individual values, higher correlation coefficients are found. The correlation coefficients between averages of sentiment scores and averages of WVS data (counted as percentages of citizens who choose a given response in WVS questionnaires) were performed for countries where such data were available. Negative correlations were found with the percentage of people declaring they are very happy or rather happy (−0.8), or for whom leisure is very important (−0.7), or for whom democracy is very good or fairly good (−0.6) – and positive correlations with the percentage of people who agree that being a housewife is just as fulfilling as working for pay (0.7), or who agree or agree strongly that men are better leaders than women (0.8), or who would prefer not to have people of other races as neighbors (0.6), or who think that having a strong leader who does not have to bother with parliament and elections is very good (0.7). These data paint a picture of positively-oriented anthems as pertaining to more conservative countries – which indeed is in line with the fact that Central/Western Asian and Eastern-European anthems are at the higher end of the positive feeling score in Fig. 1.

The above considerations may be illustrated by a few case studies. Thus, in terms of sentiment analysis, the Albanian anthem is (cf. Supporting Information and text given in previous sections) one of those with the few anthems with distinctly negative sentiment score. Indeed, the anthem repeatedly refers to negative/stressful notions such as enemy, traitor, scared, die, martyr, vanish, fight, war – though positive notions are also present (freedom, protect, live). Similarly, the Irish anthem (also listed above) makes reference to despots, slaves, woes and fighting. At the other end, the German anthem makes no reference to any of the above-mentioned notions, but rather mentions positive or constructive notions: unity, heart, fortune, flourish, blessing. Likewise, the anthem of Bosnia&Herzegovina, also with one of the highest-positive scores (cf. Supporting Information), makes no reference of enemies, fighting or suffering – but rather insists on terms such as light, mother, beautiful, proud, live, future:

You're the light of the soul // Eternal fire's flame // Mother of ours, o land of Bosnia // I belong to you // The beautiful blue sky // Of Herzegovina // In the heart are your rivers // Your mountains // Proud and famous // Land of ancestors // You shall live in our hearts // Ever more // Generations of yours // Show up as one // We go into the future // Together! // We go into the future // Together!

## 4 Conclusions

Employing an automated analysis (using the Tropes and Semantria software packages), national anthems are compared in the present study in terms of dominating notions, syntactic differences and sentiment conveyed. The identified preferred topics and the relative weights thereof (e.g., state, feeling, body, time, land, religion, family, fight, behavior, liberty) differ across continents and cultures. For instance, liberty is more common in anthems of Latin countries and almost absent in Asian ones. Feelings are less common in Latin-European and Germanic-language anthems. Religion is less common in Slavic anthems. The first-person singular “I” is essentially absent in the anthems of African countries. The sentiment score of the anthems varies across geographical regions - with an average that is essentially neutral rather than positive for Latin and Mediterranean anthems, compared to a distinctly higher positive sentiment in the anthems of Central and Western Asian, Germanic and Slavic countries.

## Production notes

### Author contribution statement

Radu Silaghi-Dumitrescu: Conceived and designed the experiments; Performed the experiments; Analyzed and interpreted the data; Contributed reagents, materials, analysis tools or data; Wrote the paper.

### Data availability statement

Data included in article/supp. material/referenced in article.

## Declaration of competing interest

The authors declare that they have no known competing financial interests or personal relationships that could have appeared to influence the work reported in this paper.

## References

[1] Abrams D. (2009). Social identity on a national scale: optimal distinctiveness and young people's self-expression through musical preference. Group Process. Intergr. Relat..

[2] Giles H., Denes A., Hamilton D.L., Hajda J.M. (2009). Striking a chord: a prelude to music and intergroup relations research. Group Process. Intergr. Relat..

[3] Lonsdale A.J., North A.C. (2009). Musical taste and ingroup favouritism. Group Process. Intergr. Relat..

[4] Dixon T.L., Zhang Y., Conrad K. (2009). Self-esteem, misogyny and afrocentricity: an examination of the relationship between rap music consumption and African American perceptions. Group Process. Intergr. Relat..

[5] Reyna C., Brandt M., Tendayi Viki G. (2009). Blame it on hip-hop: anti-rap attitudes as a proxy for prejudice. Group Process. Intergr. Relat..

[6] Rentfrow P.J., McDonald J.A., Oldmeadow J.A. (2009). You are what you listen to: young people's stereotypes about music fans. Group Process. Intergr. Relat..

[7] Rodríguez-Bailón R., Ruiz J., Moya M. (2009). The impact of music on automatically activated attitudes: Flamenco and gypsy people. Group Process. Intergr. Relat..

[8] Bensimon M. (2009). The dynamic of songs in intergroup conflict and proximity: the case of the Israeli disengagement from the Gaza strip. Group Process. Intergr. Relat..

[9] Kelen C.K. (2014).

[10] Cerulo K.A. (1989). Sociopolitical control and the structure of national symbols: an empirical analysis of national anthems. Soc. Forces.

[11] Lester D., Gunn J.F. (2011). Lyrics of national anthems and suicide rates. Psychol. Rep..

[12] Vörös V., Osváth P., Vincze O., Pusztay K., Fekete S., Ríhmer Z. (2016). Word use and content analysis of the first verses of six national anthems: a transcultural aspect of suicidal behaviour. Psychiatr. Danub..

[13] Sondermann K., Carvell T., Hyvarinen M. (2013). Interpreting the Political: New Methodologies.

[14] Abril C.R., Hebert D.G., Kertz-Welzel A. (2012). Patriotism and Nationalism in Music Education.

[15] Barnes C.D., Pomerantz A., Yashko L. (2016). Children cover your eyes: masculine honor and the role of blind patriotism in teaching national allegiance to posterity. Polit. Psychol..

[16] Kelen C.K. (2015). ‘And ever give us cause’: understanding the investments of the ‘Ur-anthem’, ‘God Save the King/Queen. Natl. Ident..

[17] Winstone N., Witherspoon K. (2016). “It's all about our great Queen”: the British National Anthem and national identity in 8-10-year-old children. Psychol. Music.

[18] Boufoy-Bastick B. (2012).

[19] Guerrini S.C., Kennedy M.C. (2009). Cross-cultural connections: an investigation of singing Canadian and American patriotic songs. Bull. Counc. Res. Music Educ..

[20] Gilboa A., Bodner E. (2009). What are your thoughts when the national anthem is playing? An empirical exploration. Psychol. Music.

[21] Kyridis A.G., Mavrikou A., Zagkos C., Golia P., Vamvakidou I., Fotopoulos N. (2009). Nationalism through state-constructed symbols: the case of national anthems. Int. J. Interdis. Soci. Sci..

[22] Lauenstein O., Murer J.S., Boos M., Reicher S.D. (2015). “Oh motherland I pledge to thee ...”: a study into nationalism, gender and the representation of an imagined family within national anthems. Nations Natl..

[23] Liao T., Zhang G., Zhang L. (2012). Social foundations of national anthems: theorizing for a better understanding of the changing fate of the national anthem of China. J. Theor. Soc. Behav..

[24] Siska K. (2016). Fear Not! Turkish nationalism and the six arrows system -A state in search of a nation. Acta Juridica Hung..

[25] Pavković A. (2020). National identity in the anthems of the states emerging from SFR Yugoslavia. Natl. Ident..

[26] Oluga S.O., Seng T., Rajoo G.S.R. (2016). Replication, evocation and revocation of linguistic sexism in translated national anthems, 3L: language, Linguistics. Literature.

[27] Rodríguez J.L. (2016). The national anthem in Warao: semiotic ground and performative affordances of indigenous language texts in Venezuela. J. Ling. Anthropol..

[28] Baghana J., Buzina E.I., Glamazda S.N., Khvesko T.V., Lazareva O.P. (2019). Literary text as knowledge format. J. Res. Appl. Ling..

[29] Kosharnaya S.A., Chumak-Zhun I.I., Plotnikova L.I., Maltseva G.Y., Boldyreva S.M. (2019). The title of a literary text as a discursive phenomenon. J. Res. Appl. Ling..

[30] Silaghi-Dumitrescu R. (2020). Topics in national anthems. J. Lang. Lit..

[31] Dodds P.S., Clark E.M., Desu S., Frank M.R., Reagan A.J., Williams J.R., Mitchell L., Harris K.D., Kloumann I.M., Bagrow J.P., Megerdoomian K., McMahon M.T., Tivnan B.F., Danforth C.M. (2015). Human language reveals a universal positivity bias. Proc. Natl. Acad. Sci. U.S.A..

[32] Mollette P., Landre A. (2014). Tropes v. 8.4.4. http://www.semantic-knowldege.com.

[33] (2019). ***, Semantria v.2010. http://www.lexanalytics.com.

[34] Miller B., Kagan K. (1997). The great powers and regional conflicts: eastern Europe and the Balkans from the post-napoleonic era to the post-cold war era. Int. Stud. Q..

[35] Silaghi-Dumitrescu R. (2018). Feudalism in modern eastern Europe. Balk. forum.

[36] David D. (2015).

[37] Blaga L. (1965).

[38] Silaghi-Dumitrescu R. (2017).

