# Supplementary material for: Trends in the texts of national anthems: A comparative study
Source: Heliyon. 2023 Aug 11;9(8):e19105. doi: 10.1016/j.heliyon.2023.e19105 (PMC10458337; doi:10.1016/j.heliyon.2023.e19105)
Supplement: Multimedia component 1 [file mmc1.docx]

Supplementary Information

Is there an “I” in “anthem”? Trends in the texts of national anthems

Radu Silaghi-Dumitrescu

*Institute for Interdisciplinary studies on Bio-Nano-Sciences, Babes-Bolyai University, Cluj-Napoca, Romania; radu.silaghi@ubbcluj.ro*

Supplementary Information

Table S1. *Correlation coefficients between percentages of word categories shown in Table 1, for various groups of anthems.*

|  | **W** | **Africa** | **Amer-Lat** | **Asia** | **Asia-CtW** | **Asia-SE** | **Eur** | **Eur-Balk** | **Eur-Ger** | **Eur-Lat** | **Eur-Slav** | **Eur-W** | **W-Medit** | **W-Engl** |
| --- | --- | --- | --- | --- | --- | --- | --- | --- | --- | --- | --- | --- | --- | --- |
| W | 1.00 | 0.98 | 0.98 | 0.98 | 0.94 | 0.96 | 0.99 | 0.89 | 0.96 | 0.95 | 0.97 | 0.98 | 0.97 | 0.98 |
| Africa | 0.98 | 1.00 | 0.96 | 0.94 | 0.88 | 0.93 | 0.95 | 0.83 | 0.94 | 0.91 | 0.94 | 0.96 | 0.94 | 0.98 |
| Amer-Lat | 0.98 | 0.96 | 1.00 | 0.95 | 0.91 | 0.93 | 0.96 | 0.90 | 0.91 | 0.92 | 0.95 | 0.95 | 0.95 | 0.96 |
| Asia | 0.98 | 0.94 | 0.95 | 1.00 | 0.98 | 0.96 | 0.99 | 0.91 | 0.95 | 0.97 | 0.96 | 0.97 | 0.97 | 0.93 |
| Asia-CtW | 0.94 | 0.88 | 0.91 | 0.98 | 1.00 | 0.90 | 0.96 | 0.91 | 0.91 | 0.95 | 0.93 | 0.94 | 0.95 | 0.88 |
| Asia-SE | 0.96 | 0.93 | 0.93 | 0.96 | 0.90 | 1.00 | 0.96 | 0.86 | 0.95 | 0.90 | 0.94 | 0.96 | 0.93 | 0.93 |
| Eur | 0.99 | 0.95 | 0.96 | 0.99 | 0.96 | 0.96 | 1.00 | 0.92 | 0.98 | 0.96 | 0.96 | 0.99 | 0.98 | 0.94 |
| Eur-Balk | 0.89 | 0.83 | 0.90 | 0.91 | 0.91 | 0.86 | 0.92 | 1.00 | 0.85 | 0.87 | 0.92 | 0.87 | 0.95 | 0.81 |
| Eur-E | 0.97 | 0.93 | 0.95 | 0.98 | 0.96 | 0.95 | 0.99 | 0.96 | 0.94 | 0.95 | 0.97 | 0.96 | 0.98 | 0.91 |
| Eur-Ger | 0.96 | 0.94 | 0.91 | 0.95 | 0.91 | 0.95 | 0.98 | 0.85 | 1.00 | 0.92 | 0.92 | 0.99 | 0.94 | 0.91 |
| Eur-Lat | 0.95 | 0.91 | 0.92 | 0.97 | 0.95 | 0.90 | 0.96 | 0.87 | 0.92 | 1.00 | 0.89 | 0.95 | 0.94 | 0.89 |
| Eur-Slav | 0.97 | 0.94 | 0.95 | 0.96 | 0.93 | 0.94 | 0.96 | 0.92 | 0.92 | 0.89 | 1.00 | 0.94 | 0.96 | 0.94 |
| Eur-W | 0.98 | 0.96 | 0.95 | 0.97 | 0.94 | 0.96 | 0.99 | 0.87 | 0.99 | 0.95 | 0.94 | 1.00 | 0.96 | 0.95 |
| W-Medit | 0.97 | 0.94 | 0.95 | 0.97 | 0.95 | 0.93 | 0.98 | 0.95 | 0.94 | 0.94 | 0.96 | 0.96 | 1.00 | 0.92 |
| W-Engl | 0.98 | 0.98 | 0.96 | 0.93 | 0.88 | 0.93 | 0.94 | 0.81 | 0.91 | 0.89 | 0.94 | 0.95 | 0.92 | 1.00 |

Table S2. *Correlation coefficients between percentages of topics shown in Table 2, for various groups of anthems.*

|  | W | Africa | Amer-Lat | Asia | Asia-CtW | Asia-SE | Eur | Eur-Balk | Eur-Ger | Eur-Lat | Eur-Slav | Eur-W | W-Medit | W-Eng |
| --- | --- | --- | --- | --- | --- | --- | --- | --- | --- | --- | --- | --- | --- | --- |
| W | 1.0 | 0.7 | 0.6 | 0.9 | 0.9 | 0.9 | 0.7 | 0.7 | 0.5 | 0.4 | 0.5 | 0.5 | 0.9 | 0.3 |
| Africa | 0.7 | 1.0 | 0.5 | 0.5 | 0.5 | 0.5 | 0.3 | 0.2 | 0.3 | 0.0 | 0.2 | 0.1 | 0.4 | 0.2 |
| Amer-Lat | 0.6 | 0.5 | 1.0 | 0.4 | 0.4 | 0.3 | 0.4 | 0.5 | 0.3 | 0.3 | 0.3 | 0.0 | 0.5 | -0.3 |
| Asia | 0.9 | 0.5 | 0.4 | 1.0 | 1.0 | 0.9 | 0.3 | 0.3 | 0.2 | 0.2 | 0.4 | 0.2 | 0.7 | 0.1 |
| Asia-CtW | 0.9 | 0.5 | 0.4 | 1.0 | 1.0 | 0.8 | 0.4 | 0.3 | 0.3 | 0.1 | 0.5 | 0.3 | 0.6 | 0.2 |
| Asia-SE | 0.9 | 0.5 | 0.3 | 0.9 | 0.8 | 1.0 | 0.4 | 0.4 | 0.1 | 0.2 | 0.3 | 0.2 | 0.8 | 0.1 |
| Eur | 0.7 | 0.3 | 0.4 | 0.3 | 0.4 | 0.4 | 1.0 | 0.8 | 0.8 | 0.5 | 0.5 | 0.9 | 0.6 | 0.3 |
| Eur-Balk | 0.7 | 0.2 | 0.5 | 0.3 | 0.3 | 0.4 | 0.8 | 1.0 | 0.6 | 0.7 | 0.2 | 0.7 | 0.7 | 0.1 |
| Eur-Ger | 0.5 | 0.3 | 0.3 | 0.2 | 0.3 | 0.1 | 0.8 | 0.6 | 1.0 | 0.4 | 0.2 | 0.9 | 0.5 | 0.1 |
| Eur-Lat | 0.4 | 0.0 | 0.3 | 0.2 | 0.1 | 0.2 | 0.5 | 0.7 | 0.4 | 1.0 | -0.2 | 0.4 | 0.7 | -0.1 |
| Eur-Slav | 0.5 | 0.2 | 0.3 | 0.4 | 0.5 | 0.3 | 0.5 | 0.2 | 0.2 | -0.2 | 1.0 | 0.2 | 0.1 | 0.2 |
| Eur-W | 0.5 | 0.1 | 0.0 | 0.2 | 0.3 | 0.2 | 0.9 | 0.7 | 0.9 | 0.4 | 0.2 | 1.0 | 0.5 | 0.5 |
| W-Medit | 0.9 | 0.4 | 0.5 | 0.7 | 0.6 | 0.8 | 0.6 | 0.7 | 0.5 | 0.7 | 0.1 | 0.5 | 1.0 | 0.0 |
| W-Eng | 0.3 | 0.2 | -0.3 | 0.1 | 0.2 | 0.1 | 0.3 | 0.1 | 0.1 | -0.1 | 0.2 | 0.5 | 0.0 | 1.0 |

**Figure S1.** Sentiment scores across groups of anthems analyzed in the present study.

**Table S3.** *Statistical data for sentiment scores across groups*.

|  | **Average** | **Weighted average** | **Standard deviation** | **Skew** |
| --- | --- | --- | --- | --- |
| Africa | 0.43 | 0.45 | 0.25 | -0.12 |
| Amer-Lat | 0.33 | 0.27 | 0.24 | -1.09 |
| Asia | 0.52 | 0.41 | 0.30 | -0.03 |
| Asia-CtW | 0.65 | 0.81 | 0.27 | 0.27 |
| Asia-SE | 0.41 | 0.35 | 0.28 | -0.56 |
| Eur | 0.48 | 0.50 | 0.39 | -0.15 |
| Eur-Balk | 0.35 | 0.32 | 0.38 | 0.88 |
| Eur-Ger | 0.64 | 0.91 | 0.25 | 0.38 |
| Eur-Lat | 0.32 | 0.15 | 0.28 | -0.08 |
| Eur-Slav | 0.60 | 0.69 | 0.31 | 0.03 |
| Eur-W | 0.46 | 0.41 | 0.37 | -0.79 |
| World | 0.44 | 0.39 | 0.33 | -0.16 |
| W-Medit | 0.25 | 0.26 | 0.33 | -0.80 |
| W-Eng | 0.33 | 0.17 | 0.27 | -1.14 |
